# Pretreatment BMI Is Associated with Aggressive Clinicopathological Features of Papillary Thyroid Carcinoma: A Multicenter Study

**DOI:** 10.1155/2017/5841942

**Published:** 2017-09-20

**Authors:** Shi-tong Yu, Wanzhi Chen, Qian Cai, Faya Liang, Debin Xu, Ping Han, Jichun Yu, Xiaoming Huang

**Affiliations:** ^1^Department of Otolaryngology, Head and Neck Surgery, Sun Yat-sen Memorial Hospital, Sun Yat-sen University, Guangzhou, Guangdong 510120, China; ^2^Department of Thyroid Surgery, The Second Affiliated Hospital of Nanchang University, Nanchang, Jiangxi 330006, China

## Abstract

**Objectives:**

The aim of the present study was to analyze the association between pretreatment body mass index (BMI) and the aggressiveness of papillary thyroid carcinoma (PTC) along with its clinical outcomes in a Chinese population with BMI classification for Asians.

**Methods:**

A retrospective, observational study was conducted on patients from two teaching hospitals in China. 1622 classical PTC patients were categorized into four groups according to BMI.

**Results:**

We found that increased BMI was associated with extrathyroidal extension, multifocality, the presence of lymph node (LN) metastasis, and advancing TNM stage in PTC patients. Furthermore, compared to patients with normal weight, those in the overweight and obese group exhibited a significantly increased risk of extrathyroidal extension, multifocality, cervical LN metastasis, and advanced TNM stage. 40 and 37 patients experienced persistent and recurrent disease, respectively. No differences regarding persistent disease or recurrence were observed among the BMI groups.

**Conclusion:**

A higher pretreatment BMI has been strongly associated with aggressive features of PTC according to the BMI classification for Asians. Obesity was not found to be associated with a greater risk of recurrence.

## 1. Introduction

During the past few decades, the prevalence of thyroid carcinoma, especially papillary thyroid carcinoma (PTC), has increased in many countries [1, 2]. However, this increase cannot be completely explained by the prevalent use of ultrasound devices (including ultrasound-guided biopsy), which facilitates early detection of small cancers [2]. An increased incidence of PTCs with larger size and later stage has been reported [3, 4], suggesting that other environmental factors may contribute to this phenomenon.

The prevalence of obesity has also increased worldwide [5], and an elevated body mass index (BMI) has been associated with an increasing incidence of several cancers including colon, renal, breast cancer, and thyroid carcinoma [6, 7]. However, for PTC, the association between increased BMI and aggressive features is not conclusive due to the conflicting results of recent reports [8–16]. In fact, the Chinese population has a different lifestyle and dietary habit compared to those in Western countries. The World Health Organization (WHO) classification of BMI for Asians is also different from that of Caucasians [17]. Previous studies, however, involved American, European, and Korean populations used the WHO classification for Europeans and Americans, not for Asians. Thus, we performed this study to determine the relationship between BMI and aggressive clinicopathological features as well as the clinical outcomes of PTC, for the first time, in a Chinese population with the WHO classification for Asians.

## 2. Methods

### 2.1. Patients

Institutional approval was obtained as well as waived informed consent. Between April 2002 and January 2013, a total of 1622 patients with classical PTC who underwent a thyroidectomy performed by the same experienced surgeons at Sun Yat-sen Memorial Hospital, Sun Yat-sen University and The Second Affiliated Hospital of Nanchang University were retrospectively reviewed in this study. The standard surgical protocol in the current study were as follows: A lobectomy was performed in patients with primary thyroid carcinoma ≤1 cm, absence of extrathyroidal extension, no abnormal lesion on the contralateral lobe, and the absence of cervical lymph node metastasis on a preoperative radiologic study. And a total thyroidectomy was performed for patients with PTC > 1 cm or with minimal extrathyroidal extension or multifocality observed in frozen pathology sections. Central neck dissection (CND) was performed routinely in all PTC patients. Lateral neck dissection was performed for patients with proven neck metastasis. Laryngeal nerves and parathyroid glands were identified and preserved in all cases, unless they were macroscopically invaded. When parathyroid glands could not be preserved in situ, they were sliced and autotransplanted into the sternocleidomastoid muscle. Hypocalcemia was defined as calcium levels <2 mmol/L (normal range, 2.10–2.65 mmol/L). Recurrent laryngeal nerve (RLN) paralysis (confirmed via fiber laryngoscopy) and hypoparathyroidism (hypocalcemia that required continuous calcium replacement therapy) were considered permanent if they had been present for more than 6 months. For each patient, the height and weight reported on the medical records were collected by surgeons or nurses and used to calculate the BMI as follows: weight in kilograms divided by height in meters squared (kg/m^2^).

Serum levels of thyroid-stimulating hormone (TSH), total cholesterol (TC), and fasting glucose at the time of admission for initial surgery were used as control variables. The TSH level was measured using an immunoradiometric assay (Roche Cobas Elecsys 600, Germany). The TC level was measured using the enzymatic colorimetric method, and the fasting glucose level was measured with a nonenzymatic method. The presence of a medical history of diabetes was also recorded.

All pathology specimens were reviewed by two experienced pathologists to confirm the diagnosis, tumor characteristics, and extent of the disease. The tumor-node-metastasis (TNM) staging system was employed based on the Union for International Cancer Control-American Joint Committee on Cancer (UICC/AJCC) 7th edition classification.

All patients were evaluated by clinical examinations, including measurements of serum thyroglobulin (Tg) and Tg antibodies and neck ultrasound imaging, every 3 months for the first 2 years and annually thereafter. When Tg levels [Tg > 1 ng/mL (total thyroidectomy) or continuous rising Tg level (lobectomy)] were significantly elevated, recurrence was determined using various methods including imaging and histological examination when necessary. If patients did not meet the criteria for remission [(1) no clinical or imaging proofs of the tumor and (2) undetectable serum Tg levels during TSH suppression and stimulation in the absence of interfering antibodies.] after initial treatment, the patient was considered to have persistent disease instead of a recurrence.

Tumor recurrence was defined as an imageable disease confirmed on cytology/biopsy that was not present at the initial presentation. Disease free survival (DFS) was defined as the time from the primary operation until the date when the patient exhibited the first recurrence.

### 2.2. Statistical Analysis

All continuous variables were reported as means ± standard deviation. The BMI was categorized according to the World Health Organization (WHO) classification for Asians: underweight (<18.5 kg/m^2^), normal weight (18.5 ≤ BMI < 23 kg/m^2^), overweight (23 ≤ BMI < 27.5 kg/m^2^), and obese (≥27.5 kg/m^2^). The normal weight group was regarded as the reference group. Patient groups were compared using Student's *t*-test, a *chi-square* test, or analysis of variance, as appropriate. Logistic regression models were used in multivariate analyses and expressed in terms of odds ratios (ORs) with 95% confidence intervals (CIs). Additionally, all ORs were adjusted for gender and age. The person-months of follow-up for each patient were accrued from the date of complete clinical remission to the date of tumor recurrence or last follow-up. Cox proportional hazard models using person-months as the underlying time metric were used to calculate a hazard ratio and 95% CI for recurrence. Disease free survival (DFS) according to BMI group was analyzed using the Kaplan–Meier method and compared among groups using the log-rank test. All statistical tests were performed with SPSS Software 19.0 (SPSS, Chicago, IL, USA). A *p* value < 0.05 was considered statistically significant.

## 3. Results

We identified 1622 patients with classical PTC who underwent surgery, as central neck dissections and lateral neck dissection were performed when appropriate. The clinical characteristics of patients according to their baseline BMI are listed in Table 1. The participants included 373 males (22.86%) and 1259 females (77.14%) with a mean age of 45.1 ± 9.8 years. The age of patients was not significantly different between groups. The rate of diabetes was identified as significantly increased (*p* < 0.001) among 4 groups with results of 2 patients (2.56%) in BMI < 18.5, 23 patients (2.78%) in 18.5 ≤ BMI < 23, 27 patients (6.52%) in 23 ≤ BMI < 27.5, and 48 (15.8%) in BMI ≥ 27.5. The rate of hyperlipidemia was found to be associated with BMI classification (*p* < 0.001) with results of 2 patients (2.56%) in BMI < 18.5, 79 patients (9.56%) in 18.5 ≤ BMI < 23, 89 patients (21.5%) in 23 ≤ BMI < 27.5, and 74 (24.34%) in BMI ≥ 27.5. The rate of hyperthyroidism, TSH levels, and number of dissected LNs was not significantly different among the four BMI groups.

### 3.1. Associations between BMI and the Clinicopathological Features of PTC

We found that increased BMI was strongly associated with extrathyroidal extension (*p* = 0.04), multifocality (*p* = 0.02), the presence of LN metastasis (*p* = 0.045), and advanced TNM stage (*p* = 0.001) in patients with PTC. No significant differences among BMI groups were observed regarding tumor size, maximum tumor size (<1 cm versus ≥1 cm), bilaterality, or distant metastasis (Table 2).

We further explored the risks of more aggressive clinicopathological features according to BMI groups (Table 3). Compared to the normal weight group, patients in the underweight group exhibited an increased OR of distant metastasis, although the difference was not statistically significant. Patients in the overweight group exhibited a significantly increased risk of extrathyroidal extension (OR = 1.32, *p* = 0.04), multifocality (OR = 1.45, *p* = 0.009), and advanced TNM stage (OR = 1.47, *p* = 0.006) compared to those in the normal weight group. Subjects in obese group exhibited a significantly greater risk of extrathyroidal extension (OR = 1.61, *p* = 0.01), multifocality (OR = 1.58, *p* = 0.02), cervical LN metastasis (OR = 1.58, *p* = 0.02), and advanced TNM stage (OR = 1.81, *p* = 0.002).

Patients in the overweight group had a greater risk of cervical LN metastasis and distant metastasis than those in the normal group, but none of these differences were statistically significant. Members of the obese group had a higher frequency of primary tumor size >1 cm, distant metastasis than those in the normal group, but none of these differences were statistically significant.

### 3.2. Association among PTC, Postoperative Complications, and Outcomes

Overall postoperative complications became more common as the BMI increased, but no significant differences were observed (Table 4). The postoperative complication rates, including hypocalcemia and RLN palsy, were not significantly different among BMI groups. Additionally, permanent complications were not associated with BMI categories.

**Figure 1:**
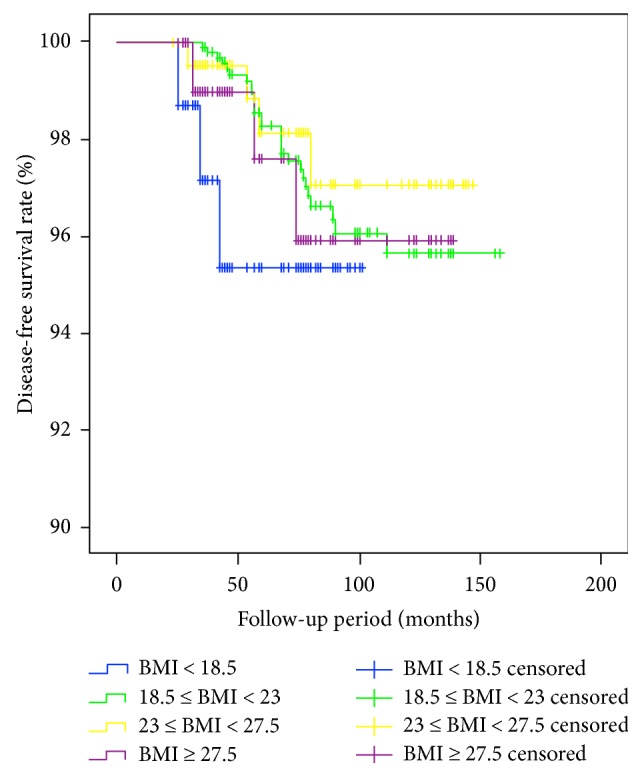
Comparison of the recurrence-free survival rate according to body mass index group. No significant differences were detected among individuals in the underweight, normal body weight, overweight, or obese groups (*p* = 0.64).

Disease status was evaluated at the end of the study after a median follow-up of 73 months (range, 25–156 months). Among the 1622 patients, 40 (2.47%) and 37 (2.28%) patients experienced persistent and recurrent disease, respectively. No differences in persistent disease were observed among the BMI groups (Table 4). Recurrence occurred at a median time of 56 months (range, 23–88 months) after complete remission. No association was found between the recurrence and BMI group for PTC patients (hazard ratio 1.132; 95% CI 0.329–1.695; *p* = 0.55). DFS was compared among groups using the log-rank test (Figure 1), but no significant differences were found (*p* = 0.64).

## 4. Discussion

Obesity has been widely studied as a risk factor for the occurrence of PTC [7, 18–20]. However, several studies have shown inconclusive results in association between BMI and aggressive clinicopathological features [7–11, 20–23]. In fact, few Chinese populations are overweight or obese compared to Western countries [17]. Previous studies have investigated the Asian population [11, 13, 24], the number of obese patients (2.6%–4.6%) were relatively smaller than studies based on Western populations (14.4%–21%), due to applied WHO classification for Caucasians to the Asian population. This difference in BMI distribution may be responsible for these conflicting results. In this study, using WHO BMI classification for Asians, our results show that BMI > 23 is also associated with the aggressiveness of PTC in the Chinese population with a similar number of obese patients (18.7%) compared to the Western countries' reports. This result confirms the general consensus that increasing BMI is positively correlated with the aggressiveness of PTC is also true in the Chinese population.

Similar to other studies [8, 12, 13], we failed to find a relationship between increased BMI and tumor persistent/recurrence in this study, even though there were differences in clinicopathological features known to be prognostic factors for PTC. One reason for explanation is that the follow-up periods in our studies and others' studies were relatively short, further studies involve longer follow-up are needed to confirm this finding. Additionally, unlike Tresallet et al. [25] who reported that an increasing BMI may increase postoperative complication rate in all PTC patients, no significant differences were observed between increasing BMI and complication rates among these patients. This finding may suggest that obesity does not influence the risk of complications or the quality of the surgery. Two experienced surgeons from two large university-teaching hospitals in China with over 400 cases of thyroidectomy per person per year may explain this. Furthermore, the dissected LN number was not different among patients demonstrating equivalent neck dissection quality.

Some biological factors may explain this obesity-cancer relationship. TSH levels have been regarded as a factor that promotes aggressiveness of PTC in obese patients [26], but the serum TSH levels were similar in all four BMI groups. Insulin-like factors and leptin have been suggested to play vital roles in thyroid carcinoma growth, and they have been associated with the aggressiveness of PTC in obese patients by increasing insulin or insulin-like growth factor levels through the PI3K/AKT and JAK2-STAT3 signaling pathways [27–29]. Hypercholesterolemia or diabetes (especially in women) may also be linked to aggressive PTC features [30]. We cannot conclude that these factors promote aggressiveness of PTC, although higher rates of diabetes and hypercholesterolemia were found in this study. Future studies will focus on the precise mechanism of this relationship to determine whether an increased BMI is a predictor of more aggressive PTC.

Our study had several limitations. First, the retrospective nature of the study was the main limitation. Second, we fail to explain the connection between an elevated BMI and DFS, maybe a longer follow-up period can explain this. Thirdly, we lacked information on other potential factors including duration of obesity, alcohol intake, and cigarette smoking. Additionally, the waist circumference, waist/hip ratio, neck circumference, and skinfold thickness were not available, and these measures may be better indexes for the assessment of obesity. A multicenter, prospective, randomized-controlled study with a longer follow-up period can be conducted in the future to address these limitations.

## 5. Conclusion

In conclusion, to our knowledge, it is the first report that we observed that excess BMI was positively associated with aggressive clinicopathological features of classical PTC in a Chinese population with the BMI classification for Asians. However, excess BMI may not influence the risk of postoperative complications, locoregional persistent disease, or recurrence. Our study provides a starting point for future studies on the potential mechanism underlying the association between obesity and the aggressiveness of PTC in a larger multicenter cohort.

## Figures and Tables

**Table 1:** Clinical characteristic of 1622 patients with PTC.

	BMI at diagnosis (kg/m^2^)				*p* value
	BMI < 18.5 *N* = 78	18.5 ≤ BMI < 23 *N* = 826	23 ≤ BMI < 27.5 *N* = 414	BMI ≥ 27.5 *N* = 304	
Gender, male/female	14/64	149/677	95/319	115/189	<0.001
Age, y, mean ± SD	43.6 ± 9.1	45.7 ± 8.9	44.5 ± 9.5	45.5 ± 9.2	0.14
Diabetes	2 (2.56%)	23 (2.78%)	27 (6.52%)	48 (15.8%)	<0.001
Hyperlipidemia	2 (2.56%)	79 (9.56%)	89 (21.5%)	74 (24.34%)	<0.001
Hyperthyroidism	8 (10.26%)	78 (9.44%)	68 (16.43%)	40 (13.16%)	0.84
TSH, mU/mL, mean ± SD	1.2 ± 1.1	1.3 ± 0.9	1.6 ± 1.2	1.5 ± 1.2	0.20
Number of LN dissected	16 ± 15	17 ± 13	19 ± 14	18 ± 17	0.39

**Table 2:** Pathological factors of 1622 PTC patients.

	BMI at diagnosis (kg/m^2^)				*p* value
	BMI < 18.5 *N* = 78	18.5 ≤ BMI < 23 *N* = 826	23 ≤ BMI < 27.5 *N* = 414	BMI ≥ 27.5 *N* = 304	
Tumor size, cm, mean ± SD	0.9 ± 0.5	1.1 ± 0.5	1.3 ± 0.6	1.3 ± 0.5	0.12
Maximum tumor size, cm					0.14
<1 cm	48 (61.54%)	486 (58.83%)	267 (64.5%)	156 (51.32%)	
≥1 cm	30 (38.46%)	340 (41.16%)	147 (35.5%)	148 (48.68%)	
Multifocality	24 (30.77%)	263 (31.84%)	167 (40.34%)	129 (42.43%)	**0.02**
Bilateriality	21 (26.92%)	174 (21.07%)	96 (23.19%)	68 (22.36%)	0.49
Extrathyroidal extension	27 (34.62%)	287 (34.75%)	170 (41.06%)	141 (46.38%)	**0.04**
N1	28 (35.9%)	314 (38.01%)	163 (39.37%)	149 (49.01%)	**0.045**
N1a	19	176	118	83	
N1b	9	138	45	66	
M1	3 (3.85%)	14 (1.69%)	11 (2.68%)	6 (1.97%)	0.65
TNM stage					**0.001**
I + II	49	525	225	150	
III + IV	29 (37.18%)	301 (36.44%)	189 (45.65%)	154 (50.66%)	

**Table 3:** Risk of aggressiveness clinicopathological features in patients with papillary thyroid cancer according to BMI group.

	BMI at diagnosis (kg/m^2^)			
	BMI < 18.5 *N* = 78	18.5 ≤ BMI < 23 *N* = 826	23 ≤ BMI < 27.5 *N* = 414	BMI ≥ 27.5 *N* = 304
Primary tumor size ≥1 cm				
Number of patients (%)	30 (38.46%)	340 (41.16%)	147 (35.5%)	148 (48.68%)
OR (95% CI)	0.90 (0.56–1.44)	Reference	0.79 (0.59–1.06)	1.33 (0.90–1.98)
*p* value	0.37		0.07	0.10
Extrathyroidal extension				
Number of patients (%)	27 (34.62%)	287 (34.75%)	170 (41.06%)	141 (46.38%)
OR (95% CI)	0.99 (0.61–1.60)	Reference	1.32 (0.98–1.76)	1.61 (1.08–2.4)
*p* value	0.54		**0.04**	**0.01**
Multifocality				
Number of patients (%)	24 (30.77%)	263 (31.84%)	167 (40.34%)	129 (42.43%)
OR (95% CI)	0.95 (0.58–1.56)	Reference	1.45 (1.08–1.94)	1.58 (1.05–2.36)
*p* value	0.48		**0.009**	**0.02**
Cervical lymph node metastasis				
Number of patients (%)	28 (35.9%)	314 (38.01%)	163 (39.37%)	149 (49.01%)
OR (95% CI)	0.91 (0.57–1.47)	Reference	1.06 (0.79–1.42)	1.57 (1.06–2.34)
*p* value	0.40		0.37	**0.02**
Distant metastasis				
Number of patients (%)	3 (3.85%)	14 (1.69%)	11 (2.68%)	2 (1.89%)
OR (95% CI)	2.27 (0.66–7.79)	Reference	1.57 (0.63–3.94)	1.09 (0.25–4.73)
*p* value	0.17		0.23	0.57
Advanced TNM stage				
Number of patients (%)	29 (37.18%)	301 (36.44%)	189 (45.65%)	154 (50.66%)
OR (95% CI)	1.03 (0.64–1.66)	Reference	1.47 (1.10–1.96)	1.81 (1.23–2.70)
*p* value	0.50		**0.006**	**0.002**

**Table 4:** Postoperative complications and persistent disease in patients with PTC.

	BMI at diagnosis (kg/m^2^)				*p* value
	BMI < 18.5 *N* = 78	18.5 ≤ BMI < 23 *N* = 826	23 ≤ BMI < 27.5 *N* = 414	BMI ≥ 27.5 *N* = 304	
Postoperative complications^∗^	4 (5.13%)	49 (5.93%)	27 (6.52%)	29 (9.54%)	0.43
Hypocalcaemia	3 (3.85%)	41 (4.96%)	26 (6.28%)	27 (8.89%)	0.42
Transient	3	39	26	26	
Permanent	0	2	0	1	
RLN injury	2 (2.56%)	18 (2.2%)	11 (2.66%)	8 (2.63%)	0.89
Transient	2	17	11	8	
Permanent	0	1	0	0	
Abscess	1	2	0	2	0.19
Postoperative bleeding	0	1	0	1	0.10
Overall postoperative permanent complications	0	3	0	1	0.42
Persistent disease	3 (3.85%)	18 (2.18%)	11 (2.66%)	8 (2.63%)	0.37

^∗^Some patients may have more than 1 complication.
